# Theoretical Model of Droplet Wettability on a Low-Surface-Energy Solid under the Influence of Gravity

**DOI:** 10.1155/2014/647694

**Published:** 2014-01-08

**Authors:** Yukihiro Yonemoto, Tomoaki Kunugi

**Affiliations:** ^1^Priority Organization for Innovation and Excellence, Kumamoto University, 2-39-1, Kurokami, Chuo-ku, Kumamoto-shi, Kumamoto 860-8555, Japan; ^2^Department of Nuclear Engineering, Kyoto University, C3-d2S06, Kyoto Daigaku-Katsura, Nishikyo-ku, Kyoto 615-8540, Japan

## Abstract

The wettability of droplets on a low surface energy solid is evaluated experimentally and theoretically. Water-ethanol binary mixture drops of several volumes are used. In the experiment, the droplet radius, height, and contact angle are measured. Analytical equations are derived that incorporate the effect of gravity for the relationships between the droplet radius and height, radius and contact angle, and radius and liquid surface energy. All the analytical equations display good agreement with the experimental data. It is found that the fundamental wetting behavior of the droplet on the low surface energy solid can be predicted by our model which gives geometrical information of the droplet such as the contact angle, droplet radius, and height from physical values of liquid and solid.

## 1. Introduction

Wetting phenomena are widely important in industrial and chemical fields. For example, the wettability of droplets or bubbles on solid surface affects heat transfer, which affects efficient heat exchange [1, 2]. Recently, wettability phenomena on the microscopic scale have received attention because they are relevant for optimizing the surface design of microchannels [3] that aid realization of highly efficient chemical reactions. While studies have been performed for more than 100 years, we do not yet have a full and complete understanding of wetting because this phenomenon exhibits complex behavior such as contact angle hysteresis and does not necessarily obey Young's relation [4].

Recent research has focused on simulation of the dynamic behavior of droplets or bubbles on solid surfaces [5–7] using nonlinear differential equations. The contact angle is treated as a boundary condition at a three-phase line on a solid surface. To treat the dynamic contact angle, semiempirical equations are mainly used to determine the contact angle [8–10]. On the other hand, the contact angle hysteresis also appears in a phase change of a droplet on a solid surface. As the droplet evaporates, it exhibits three principle stages of wetting behavior. Initially, the contact angle changes while the droplet radius remains constant. Then, the droplet radius changes at a constant contact angle. Finally, both the droplet radius and the contact angle change simultaneously. These processes depend on the kind of solid [11]. Especially, in the second process, a stick-slip motion of the contact line occurs. Shanahan and Sefiane studied the stick-slip phenomena experimentally and theoretically [12]. They observed an evaporating ethanol droplet, and presented a simple theoretical model from a thermodynamic perspective. In their study, assuming a droplet of a spherical cap, the Gibbs free energy and volume evaporation rate were evaluated. Finally, they qualitatively explain the stick-slip behavior by invoking a pinning time and a slip time and introducing the concept of free energy barriers at a contact line.

The wettability of droplets on solid surfaces has been studied from various theoretical and experimental viewpoints and have revealed other droplet hystereses, such as a size dependency of the contact angle [13]. However, the detailed mechanism of this behavior is still unknown. The contact angle hysteresis resulting from various physical characteristics makes it difficult to correctly evaluate wetting phenomena using the contact angle only. Generally, droplet shape is predicted using the differential equation on the basis of Young-Laplace law [14, 15]. This method is applicable for the prediction of the shape of droplet providing some geometrical information such as contact radius from a given image of a droplet. However, the wetting phenomena of droplet exhibit various behaviors depending on kinds of the liquid and solid. The interaction between the liquid and the solid in addition to the gravity effect determines the size of the contact radius, and height, and so forth. Therefore, in order to understand the physics of the peculiar phenomena such as droplet hysteresis, discussions about the fundamental droplet behavior are important and necessary to construct a basic analytical model using physical properties of liquid and solid for the wetting phenomena.

In the present study, the wetting behavior of a droplet on a solid surface under the influence of gravity is considered theoretically and experimentally. In the theory, the adhesion energy, including the effects of gravity, is evaluated as the droplet volume changes. Wetting is determined by interactions between the liquid and the solid. Thus, the inclusion of solid properties in the theoretical model is critically important for developing a general framework. Thus, in our model, the critical surface tension (*σ*_*c*_) is chosen for evaluation of the solid surface energy from an engineering point of view. Finally, an analytical equation that can treat the droplet behavior under the gravity condition is derived. In the experimental approach, water-ethanol binary mixture drops of several volumes are used, where the volume ratio of the ethanol is in the range from 0 to 100%. The substrate is a low surface energy solid (silicone rubber). The contact angle, droplet radius, and height are experimentally measured for each droplet volume. When comparing the theoretical results with the experimental data, the theory shows good agreement with the experimental data of the relationships among *R*, *σ*_*lg*_, and *θ*. In addition, our model can give geometrical information of the droplet on the low surface energy solid surface such as *R*, *h*, and *θ* from the physical properties of *σ*_*lg*_, *σ*_*c*_ and *ρ*_*l*_.

## 2. Experiment

In this study, we used water-ethanol binary mixtures to investigate the wetting behavior on solid surfaces [16–19]. The volume ratio was varied from 0 to 100% ethanol. Specifically, we used ultrapure water (*σ*_*lg*_ = 0.0719 J m^−2^) (Wako Pure Chemical Industries, Ltd.), four kinds of binary mixtures (*σ*_*lg*_ = 0.0570, 0.0384, 0.0312, and 0.0274 J m^−2^), and pure ethanol (0.0211 J m^−2^) (Nacalai Tesque, Inc., 99.5% pure). The surface energy of the liquids was measured using a DM300 (Kyowa Interface Science Co., Ltd.). Droplets were set on a solid surface using a micropipette. For each liquid, droplet volumes of 5, 10, 20, and 40 *μ*L were used. The main observables measured in the experiment were the droplet radius, height, and contact angle. The silicon rubber chosen as the solid material for all our studies was rinsed using ethanol and purified water before each experiment and then dried. In the experiment, a test section is covered with a clean booth in which the temperature and relative humidity were kept in the range of 20.0–23.0°C and 50.0–55.0%, respectively. An image of the droplet is captured using a CCD camera (Yashima Optical Co., Ltd.). A maximum error for the size measurement is ±14.5 *μ*m/pixel.

## 3. Theory

**Figure 1:**
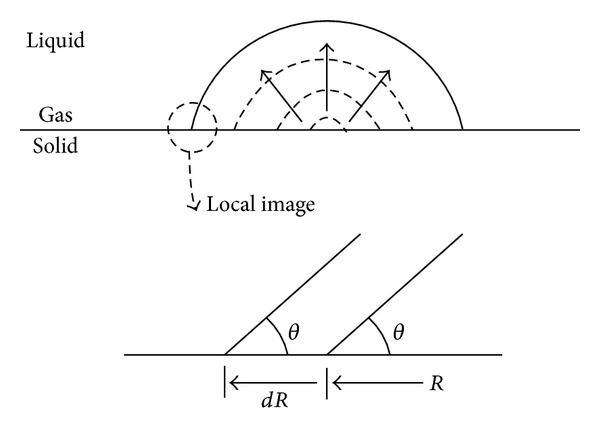
Mathematical image of the droplet.

Consider a situation where a droplet is slowly deposited on a solid surface. In a mathematical image, the volume change of the droplet can be represented as shown in Figure 1. The contact line moves with an infinitesimal length in a circumferential direction due to the volume increase when an infinitesimal volume change is considered. On the basis of this concept, the change in energy as a result of this volume increase can be mathematically expressed as follows:

(1) dEg=ρlgd(z−V).

In this equation, *ρ*_*l*_ is the density of the liquid (kg m^−3^), *g* is the gravitational acceleration (m s^−2^), $\overset{-}{z}$ is the gravity point (m), and *V* is the volume of droplet (m^3^). Then, the change in the work-energy that results from displacement of the contact line is expressed as follows:

(2) dEhorizontal=2πR(−σsg)dR+2πR(σsl+σlg)dR.

By equating (1) and (2) and integrating the relation, the following equation is obtained:

(3) $$\begin{array}{c} \overset{\overset{-}{z}V}{\underset{0}{\int }}\rho_{l}g\text{d}\left(\overset{-}{z}V\right)=\overset{R}{\underset{0}{\int }}2\pi R\left(-\sigma_{sg}+\sigma_{sl}+\sigma_{lg}\right)\text{d}R; \end{array}$$

then,

(4) $$\begin{array}{c} \rho_{l}g\overset{-}{z}V=\pi R^{2}\sigma_{lg}\left(1-\cos\theta \right). \end{array}$$

In the derivation of (4), the Young equation is applied. A similar equation is derived by de Gennes et al. [20]. Here, in our model, an additional work component is considered in (3). It is the work-energy in the vertical direction at the contact line during the change in the height of droplet. This energy should be also considered as the energy change during the contact line displacement, which is modeled as follows:

(5) $$\begin{array}{c} E_{\text{vertical}}=-\overset{L}{\underset{0}{\int }}\left[\overset{h}{\underset{0}{\int }}f_{\text{vert}}\frac{h-z}{h}\text{d}z\right]\text{d}s, \end{array}$$

where *f*_vert_ is *σ*_*lg*_sin*θ*, the capillary force in the vertical direction at the contact line. *L* is 2*πR*, the length of the contact circle, *s* and *z* are the parameters along the contact circle and in the direction of the droplet height, respectively, and *h* is the droplet height. This equation is based on the concept that the adhesion energy in the vertical direction can be estimated as the work that corresponds to the height of the droplet at most. Finally, by adding (5) into the right-hand side of (3), the following equation is obtained:

(6) ρlgz−V=πR2σlg(1−cosθ)−πRhσlgsinθ.

For the sake of simplicity, a simple height average is applied at the gravity point ($\overset{-}{z}=h/2$):

(7) ρlghV2=πR2σlg(1−cosθ)−πRhσlgsinθ.

This equation is the base relation for our analysis. Then, in our model, the solid surface energy is evaluated based on the concept of Zisman. The basic relation is the following equation:

(8) cosθ=−1+2σcσlg.

This relation predicts the contact angle of binary liquid mixtures on low surface energy solids [21]. Equation (7) can be rearranged using (10) as follows:

(9) $$\begin{array}{c} \frac{\rho_{l}ghV}{2}=2\pi R^{2}\left(\sigma_{lg}-\sigma_{c}\right)-2\pi Rh\sqrt{\sigma_{c}\left(\sigma_{lg}-\sigma_{c}\right)}. \end{array}$$

In our model, (7) or (9) is a basic equation for the prediction of the droplet behavior.

## 4. Result and Discussion

**Figure 2:**
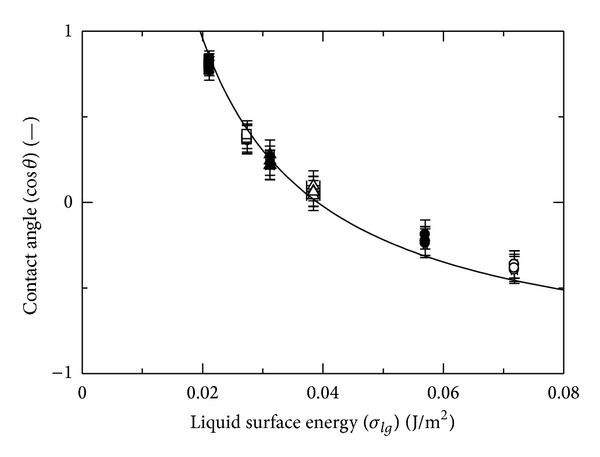
Relationship between the liquid surface energy and contact angle for different drop volumes from 5 *μ*L to 40 *μ*L.

The critical surface tension, which is needed in the theoretical analysis, is obtained from the relationship between the surface energy of liquid and the contact angle as shown in Figure 2. The solid line is fitted using (8). From this result, the critical surface tension is estimated to be 0.01956 J m^−2^, which is of the same order as that reported previously [22].

To compare the theoretical and experimental data, a relationship between the liquid density and its surface energy is required. Such a relation is difficult to obtain theoretically. Thus, an empirical equation can be used to describe this relation as follows:

(10) $$\begin{array}{c} \rho_{l}=\rho_{w}+\frac{\rho_{e}-\rho_{w}}{100}\left(A+\frac{\text{ex}{\text{p}}^{2}\left(B\right)}{\sigma_{lg}^{2}}\right), \end{array}$$

where the values of *ρ*_*w*_ = 998.2 kg m^−3^ and *ρ*_*e*_ = 789.2 kg m^−3^ are the water and ethanol densities at room temperature, respectively. *A* and *B* are arbitrary fitting parameters. In the present study, *A* and *B* were found to be −9.65 and −1.50, respectively. Thus, the liquid density (*ρ*_*l*_) in (7) or (9) is calculated using (10).

**Figure 3:**
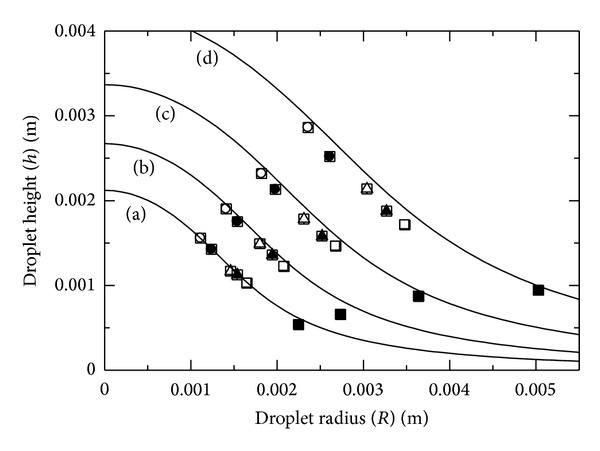
Relationship between droplet radius and height. The solid lines are (a) 5, (b) 10, (c) 20, and (d) 40 *μ*L, respectively. The white circles, black circles, white triangles, black triangles, white squares, and black squares are experimental data of 0.071, 0.0570, 0.0384, 0.0312, 0.0274, and 0.0211 J m^−2^, respectively.

Figure 3 shows the measured fundamental relationship between the radius and height of the droplets. The solid lines are evaluated by the following relation (volume of spherical cap):

(11) $$\begin{array}{c} V_{0}=\pi h\left(\frac{h^{2}}{6}+\frac{R^{2}}{2}\right). \end{array}$$

Data for each liquid surface energy in Figure 3 are relatively well fit to (11), although the experimental data depart from the solid line due to the effect of gravity as the droplet volume increases. Here, regarding the droplet volume (*V*), (11) is used as an approximation (*V* ≈ *V*_0_) for the volume in (7) or (9) because of good agreement with the experimental data as shown in Figure 1. Thus, in the analysis, (7) or (9), using (8), and (10) are numerically solved with the bisection method.

**Figure 4:**
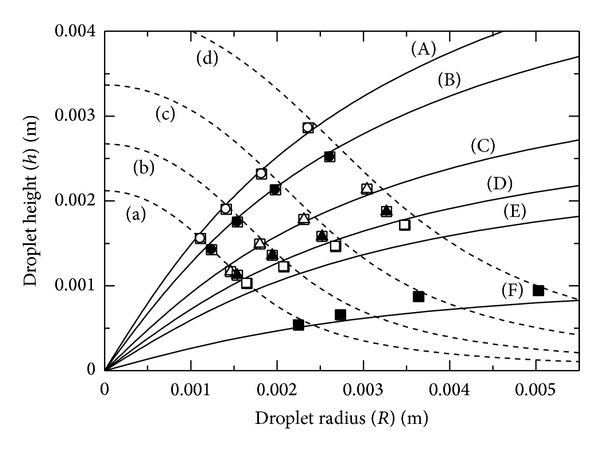
Relationship between droplet height and radius: (a) 5, (b) 10, (c) 20, and (d) 40 *μ*L are analytical solutions; (A) 0.0718, (B) 0.057, (C) 0.0384, (D) 0.0312, (E) 0.0274, and (F) 0.0211 J m^−2^ are theoretical lines.

Figure 4 shows the relationship between droplet radius (*R*) and height (*h*) for various droplet volumes. The dashed lines labeled (a)–(d) are the analytical results of (11) for volumes of 5, 10, 20, and 40 *μ*L, respectively. The solid lines labeled (A)–(F) are the theoretical traces for 0.0719, 0.057, 0.0384, 0.0312, 0.0274, and 0.0211 J m^−2^, respectively. Focusing on the cases of constant surface energy, the experimental data shows good agreement with each theoretical line. As the droplet radius (volume) increases, the droplet height also increases, but the gradient of d*h*/d*R* gradually decreases. The relationship between *R* and *h* is nonlinear. These results indicate the validity of our model with respect to the gravity effect.

**Figure 5:**
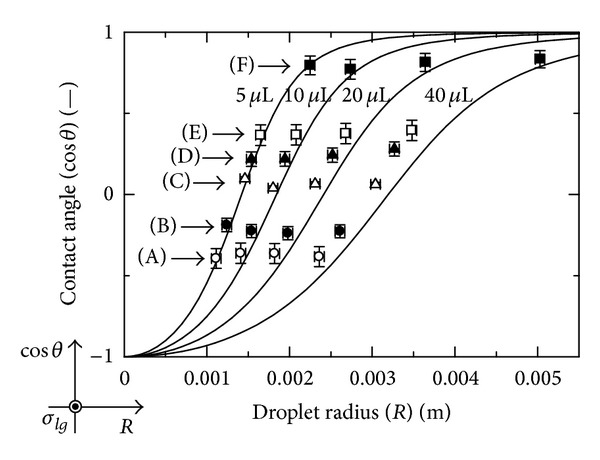
Relationship between droplet radius and contact angle.

The relationship between droplet radius (*R*) and contact angle (*θ*) for some constant volumes is shown in Figure 5. The solid lines are calculated from the theoretical equations. Experimental data shows good agreement with each theoretical line. In each theoretical result at a constant volume, the contact angle approaches zero as the surface energy of liquid decreases. The droplet radius takes on large values when the contact angle approaches zero. This is indicative of complete wetting. On the other hand, at a constant liquid surface energy, the contact angle is nearly constant from the (A) to (F) cases in the present study.

**Figure 6:**
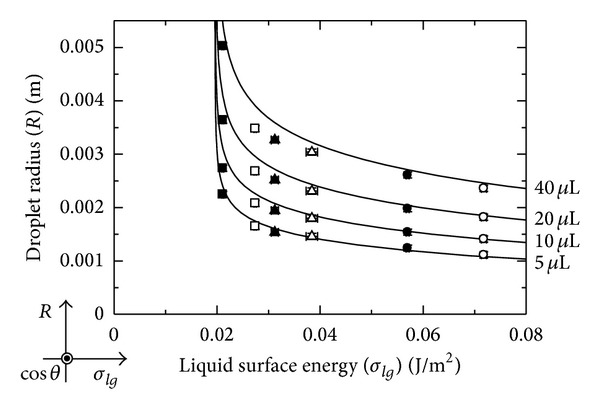
Relationship between the liquid surface energy and droplet radius.

In Figure 6, the relationship between the liquid surface energy (*σ*_*lg*_) and the droplet radius (*R*) is shown. The solid lines are the theoretical results. At a constant droplet volume, as the liquid surface energy decreases, the droplet radius increases and takes on large values near the critical surface tension. This tendency also indicates complete droplet wetting, as shown in Figure 5. However, in an actual system, the droplet radius will approach a finite value, because volume conservation must hold, beyond which the liquid cannot spread even if the liquid surface energy is nearly the same value as the critical surface tension.

From the results above, droplet behavior on a low surface energy solid is evaluated through examination of the relationships between the droplet radius (*R*), contact angle (*θ*), and liquid surface energy (*σ*_*lg*_). Constructing a three-dimensional space of *R*-*σ*_*lg*_-cos *θ*, it is found that the Zisman plot maps well to the *σ*_*lg*_-cos *θ* plane surface in *R*-*σ*_*lg*_-cos *θ* space. Although wetting phenomena are related to various physical characteristics such as fluid flow, surface micro-structure, temperature, and humidity, in the present study, we are able to predict the static droplet behavior on the low surface energy solid by the use of physical parameters of liquid and solid such as *ρ*_*l*_, *σ*_*lg*_, and *σ*_*c*_.

## 5. Conclusion

In this paper, the wettability of binary mixture droplets on silicone rubber, which is a low surface energy solid, is investigated. In the experiment, the relationship between the surface tension and geometric configurations such as contact angle and droplet radius are evaluated. Theoretically, analytical equations are derived considering effects due to gravity and solid surface properties. The derived equations show good agreement with the experimental data for some relationships among the surface tension, droplet size, and contact angle. The results indicate that liquid wettability on low surface energy solids could be predicted by our model which gives geometrical information of droplet from the physical parameters such as the density of liquid, liquid surface energy, and the critical surface tension. Our model may serve as a basis for including complex contact angle hysteresis wetting phenomena within evaluations of droplet behavior on solid surfaces.
